# 5,6-Diphenyl-3-(3-pyrid­yl)-1,2,4-triazine

**DOI:** 10.1107/S1600536810002187

**Published:** 2010-01-23

**Authors:** Fang-Fang Jian, Ping Ren

**Affiliations:** a New Materials and Function Coordination Chemistry Laboratory, Qingdao University of Science and Technology, Qingdao 266042, People’s Republic of China

## Abstract

In the mol­ecule of the title compound, C_20_H_14_N_4_, the triazine ring is attached to two phenyl rings and one pyridine ring. In the crystal, mol­ecules are linked by inter­molecular C—H⋯N hydrogen bonds. The crystal packing is also stabilized by C—H⋯π inter­actions.

## Related literature

For applications of substituted 1,2,4-triazines, see: Denecke *et al.* (2005 ▶); Maheshwari *et al.* (2006 ▶): Zhao *et al.* (2003 ▶).
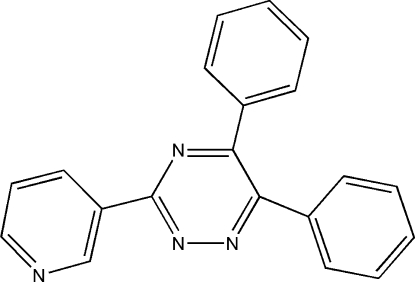


## Experimental

### 

#### Crystal data

C_20_H_14_N_4_
*M* *_r_* = 310.35
Monoclinic, 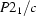

*a* = 14.4775 (16) Å
*b* = 7.0923 (8) Å
*c* = 18.5786 (15) Å
β = 125.587 (6)°
*V* = 1551.3 (3) Å^3^
*Z* = 4
Mo *K*α radiation
μ = 0.08 mm^−1^
*T* = 293 K
0.31 × 0.28 × 0.26 mm

#### Data collection

Enraf–Nonius CAD-4 diffractometer
9802 measured reflections
3770 independent reflections
2184 reflections with *I* > 2σ(*I*)
*R*_int_ = 0.033
3 standard reflections every 200 reflections  intensity decay: none

#### Refinement

*R*[*F* ^2^ > 2σ(*F* ^2^)] = 0.048
*wR*(*F* ^2^) = 0.125
*S* = 1.04
3770 reflections
218 parameters
H-atom parameters constrained
Δρ_max_ = 0.16 e Å^−3^
Δρ_min_ = −0.15 e Å^−3^

### 

Data collection: *CAD-4 Software* (Enraf–Nonius, 1989 ▶); cell refinement: *CAD-4 Software*; data reduction: *NRCVAX* (Gabe *et al.*, 1989 ▶); program(s) used to solve structure: *SHELXS97* (Sheldrick, 2008 ▶); program(s) used to refine structure: *SHELXL97* (Sheldrick, 2008 ▶); molecular graphics: *SHELXTL/PC* (Sheldrick, 2008 ▶); software used to prepare material for publication: *WinGX* (Farrugia, 1999 ▶).

## Supplementary Material

Crystal structure: contains datablocks global, I. DOI: 10.1107/S1600536810002187/hg2609sup1.cif

Structure factors: contains datablocks I. DOI: 10.1107/S1600536810002187/hg2609Isup2.hkl

Additional supplementary materials:  crystallographic information; 3D view; checkCIF report

## Figures and Tables

**Table 1:** Hydrogen-bond geometry (Å, °) *Cg*1 and *Cg*2 are the centroids of the N4,C16–C20 and C1–C6 rings, respectively.

*D*—H⋯*A*	*D*—H	H⋯*A*	*D*⋯*A*	*D*—H⋯*A*
C20—H20*A*⋯N3	0.93	2.49	2.824 (4)	102
C13—H13*A*⋯*Cg*1^i^	0.93	3.49	3.345 (4)	91
C19—H19*A*⋯*Cg*2^ii^	0.93	3.67	3.109 (4)	121

Symmetry codes: (i) 
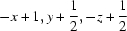
; (ii) 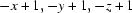.

**Fig. 1.**
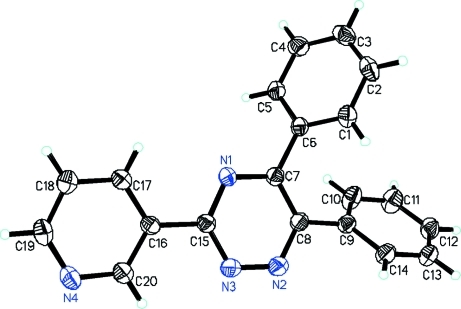
The molecular structure of the title compound with the atom-labeling scheme. Displacement ellipsoids are drawn at the 30% probability level.

## supplementary crystallographic information

### Comment

The increasing interest in the chemistry of substituted 1,2,4-triazine is due to their various applications: commercial dyes, herbicides (Zhao *et al.*, 2003), antiviral, antitumor drug (Maheshwari *et al.*, 2006) and selective extracting agents in the separation of lanthanides and actinides in the management of nuclear wastes (Denecke *et al.*, 2005). The title compound belongs to the family of these compounds. We have synthesized the title compound and describe its structure here.

In the title compound, the bond lengths and angles are generally normal. The dihedral angles between triazine ring(p1) with the pyridine ring (p2), C1—C6 (p3) and C9—C14 (p4) phenyl rings are 2.94 (2)°, 53.35 (2)° and 50.43 (2)°, respectively. There exist intermolecular C—H···N hydrogen bond and C—H···π supramolecular interactions in the crystal lattice. The donor and acceptor distance is 2.8235Å for C20—H20A···N3. In addition, there are obvious intermolecular C—H···π interactions between C13—H13A and pyridine ring (*Cg*(2)), C19—H19A and C1—C6 phenyl ring (*Cg*(3)). In the solid state, all above intermolecular interactions in the title compound stabilize the crystal packing structure.

### Experimental

To a mixture of (3-pyridylcarbonyl)hydrazine (0.828 g, 6 mmol) and benzil (1.26 g, 6 mmol) was added ammonium acetate (4.62 g, 0.06 mol) and 1 ml of acetic acid. The mixture was heated by conventional microwave oven for 5 min at 453 K. Upon rapid cooling of the reaction vessel to 313 K, a yellow precipitate formed, which was washed with water to afford the title compound (yield 45.3%). Single crystals suitable for X-ray measurements were obtained by recrystallization from ethanol at room temperature for three days.

### Refinement

H atoms were positioned geometrically and allowed to ride on their parent atoms, with C—H distances of 0.93–0.96 Å, respectively, and with *U*_iso_(H) = 1.2*U*_eq_ of the parent atoms.

### Crystal data

**Table:**

C_20_H_14_N_4_	*F*(000) = 648
*M**_r_* = 310.35	*D*_x_ = 1.329 Mg m^−^^3^
Monoclinic, *P*2_1_/*c*	Melting point: 444 K
Hall symbol: -P 2ybc	Mo *K*α radiation, λ = 0.71073 Å
*a* = 14.4775 (16) Å	Cell parameters from 25 reflections
*b* = 7.0923 (8) Å	θ = 1.7–28.3°
*c* = 18.5786 (15) Å	µ = 0.08 mm^−^^1^
β = 125.587 (6)°	*T* = 293 K
*V* = 1551.3 (3) Å^3^	Block, yellow
*Z* = 4	0.31 × 0.28 × 0.26 mm

### Data collection

**Table:**

Enraf–Nonius CAD-4 diffractometer	*R*_int_ = 0.033
Radiation source: fine-focus sealed tube	θ_max_ = 28.3°, θ_min_ = 1.7°
graphite	*h* = −16→19
ω scans	*k* = −7→9
9802 measured reflections	*l* = −24→20
3770 independent reflections	3 standard reflections every 200 reflections
2184 reflections with *I* > 2σ(*I*)	intensity decay: none

### Refinement

**Table:**

Refinement on *F*^2^	Secondary atom site location: difference Fourier map
Least-squares matrix: full	Hydrogen site location: inferred from neighbouring sites
*R*[*F*^2^ > 2σ(*F*^2^)] = 0.048	H-atom parameters constrained
*wR*(*F*^2^) = 0.125	*w* = 1/[σ^2^(*F*_o_^2^) + (0.0536*P*)^2^] where *P* = (*F*_o_^2^ + 2*F*_c_^2^)/3
*S* = 1.03	(Δ/σ)_max_ = 0.001
3770 reflections	Δρ_max_ = 0.16 e Å^−^^3^
218 parameters	Δρ_min_ = −0.15 e Å^−^^3^
0 restraints	Extinction correction: *SHELXL97* (Sheldrick, 2008), Fc^*^=kFc[1+0.001xFc^2^λ^3^/sin(2θ)]^-1/4^
Primary atom site location: structure-invariant direct methods	Extinction coefficient: 0.0105 (17)

### Special details

**Table:**

Geometry. All e.s.d.'s (except the e.s.d. in the dihedral angle between two l.s. planes) are estimated using the full covariance matrix. The cell e.s.d.'s are taken into account individually in the estimation of e.s.d.'s in distances, angles and torsion angles; correlations between e.s.d.'s in cell parameters are only used when they are defined by crystal symmetry. An approximate (isotropic) treatment of cell e.s.d.'s is used for estimating e.s.d.'s involving l.s. planes.
Refinement. Refinement of *F*^2^ against ALL reflections. The weighted *R*-factor *wR* and goodness of fit *S* are based on *F*^2^, conventional *R*-factors *R* are based on *F*, with *F* set to zero for negative *F*^2^. The threshold expression of *F*^2^ > σ(*F*^2^) is used only for calculating *R*-factors(gt) *etc*. and is not relevant to the choice of reflections for refinement. *R*-factors based on *F*^2^ are statistically about twice as large as those based on *F*, and *R*- factors based on ALL data will be even larger.

### Fractional atomic coordinates and isotropic or equivalent isotropic displacement parameters (Å^2^)

**Table:**

	*x*	*y*	*z*	*U*_iso_*/*U*_eq_	
N1	0.40833 (10)	0.74465 (17)	0.84881 (7)	0.0422 (3)	
N2	0.48148 (11)	0.5785 (2)	0.75648 (8)	0.0554 (4)	
N3	0.55312 (11)	0.5984 (2)	0.84483 (8)	0.0546 (4)	
C20	0.69841 (13)	0.6021 (2)	1.03161 (9)	0.0505 (4)	
H20A	0.7215	0.5456	0.9993	0.061*	
C1	0.19450 (14)	0.9625 (2)	0.65096 (9)	0.0512 (4)	
H1B	0.2415	0.9897	0.6334	0.061*	
C2	0.09076 (15)	1.0535 (2)	0.61123 (10)	0.0598 (5)	
H2B	0.0688	1.1431	0.5674	0.072*	
C3	0.02025 (14)	1.0123 (3)	0.63613 (10)	0.0608 (5)	
H3B	−0.0496	1.0727	0.6087	0.073*	
C4	0.05291 (13)	0.8823 (2)	0.70138 (10)	0.0555 (4)	
H4B	0.0050	0.8541	0.7180	0.067*	
C5	0.15676 (12)	0.7931 (2)	0.74260 (9)	0.0475 (4)	
H5A	0.1793	0.7073	0.7879	0.057*	
C6	0.22752 (12)	0.8308 (2)	0.71683 (9)	0.0410 (4)	
C7	0.33957 (11)	0.7350 (2)	0.76126 (8)	0.0398 (4)	
C8	0.37453 (12)	0.6390 (2)	0.71427 (9)	0.0430 (4)	
C9	0.29850 (13)	0.5980 (2)	0.61817 (9)	0.0437 (4)	
C10	0.19430 (15)	0.5132 (3)	0.58174 (10)	0.0625 (5)	
H10A	0.1710	0.4833	0.6176	0.075*	
C11	0.12424 (16)	0.4725 (3)	0.49224 (11)	0.0692 (5)	
H11A	0.0546	0.4141	0.4684	0.083*	
C12	0.15742 (15)	0.5181 (2)	0.43853 (10)	0.0581 (5)	
H12A	0.1102	0.4912	0.3783	0.070*	
C13	0.25980 (15)	0.6030 (2)	0.47375 (10)	0.0578 (5)	
H13A	0.2819	0.6354	0.4373	0.069*	
C14	0.33114 (14)	0.6412 (2)	0.56364 (10)	0.0531 (4)	
H14A	0.4016	0.6964	0.5874	0.064*	
C15	0.51278 (12)	0.6718 (2)	0.88740 (9)	0.0410 (4)	
C16	0.59002 (12)	0.6778 (2)	0.98515 (9)	0.0400 (4)	
C17	0.55691 (13)	0.7604 (2)	1.03439 (9)	0.0506 (4)	
H17A	0.4845	0.8112	1.0062	0.061*	
C18	0.63165 (14)	0.7668 (2)	1.12525 (10)	0.0569 (5)	
H18A	0.6111	0.8231	1.1592	0.068*	
C19	0.73715 (14)	0.6882 (2)	1.16431 (10)	0.0575 (5)	
H19A	0.7876	0.6932	1.2257	0.069*	
N4	0.77171 (11)	0.6048 (2)	1.11941 (8)	0.0585 (4)	

### Atomic displacement parameters (Å^2^)

**Table:**

	*U*^11^	*U*^22^	*U*^33^	*U*^12^	*U*^13^	*U*^23^
N1	0.0368 (7)	0.0495 (8)	0.0368 (6)	0.0013 (6)	0.0193 (5)	−0.0009 (6)
N2	0.0498 (9)	0.0713 (10)	0.0462 (8)	0.0062 (7)	0.0285 (7)	−0.0046 (7)
N3	0.0434 (8)	0.0729 (10)	0.0452 (8)	0.0091 (7)	0.0244 (7)	−0.0038 (7)
C20	0.0439 (9)	0.0556 (10)	0.0456 (9)	0.0041 (7)	0.0223 (8)	0.0010 (8)
C1	0.0526 (10)	0.0574 (10)	0.0405 (8)	−0.0012 (8)	0.0254 (7)	0.0024 (8)
C2	0.0567 (11)	0.0541 (11)	0.0424 (9)	0.0059 (8)	0.0138 (8)	0.0065 (8)
C3	0.0386 (9)	0.0666 (12)	0.0519 (10)	0.0081 (8)	0.0119 (8)	−0.0059 (9)
C4	0.0402 (9)	0.0705 (12)	0.0524 (10)	−0.0002 (8)	0.0249 (8)	−0.0068 (9)
C5	0.0436 (9)	0.0577 (10)	0.0378 (8)	0.0035 (7)	0.0217 (7)	0.0029 (7)
C6	0.0372 (8)	0.0486 (9)	0.0319 (7)	−0.0006 (7)	0.0171 (6)	−0.0040 (6)
C7	0.0375 (8)	0.0436 (9)	0.0371 (8)	−0.0029 (6)	0.0210 (7)	−0.0006 (6)
C8	0.0421 (8)	0.0484 (9)	0.0400 (8)	−0.0013 (7)	0.0248 (7)	−0.0011 (7)
C9	0.0477 (9)	0.0442 (9)	0.0414 (8)	0.0003 (7)	0.0271 (7)	−0.0027 (7)
C10	0.0674 (12)	0.0758 (13)	0.0490 (10)	−0.0227 (10)	0.0366 (9)	−0.0126 (9)
C11	0.0671 (12)	0.0810 (14)	0.0546 (11)	−0.0240 (10)	0.0326 (10)	−0.0166 (9)
C12	0.0664 (12)	0.0608 (11)	0.0397 (9)	0.0004 (9)	0.0267 (8)	−0.0065 (8)
C13	0.0681 (12)	0.0670 (12)	0.0479 (10)	0.0031 (9)	0.0392 (9)	0.0005 (8)
C14	0.0526 (10)	0.0626 (11)	0.0492 (9)	−0.0026 (8)	0.0325 (8)	−0.0038 (8)
C15	0.0363 (8)	0.0439 (9)	0.0407 (8)	0.0005 (7)	0.0211 (7)	−0.0006 (7)
C16	0.0349 (8)	0.0403 (8)	0.0420 (8)	−0.0022 (6)	0.0206 (7)	−0.0005 (7)
C17	0.0409 (9)	0.0596 (10)	0.0455 (9)	0.0058 (7)	0.0220 (7)	0.0009 (8)
C18	0.0576 (11)	0.0638 (11)	0.0452 (9)	0.0006 (9)	0.0276 (8)	−0.0052 (8)
C19	0.0502 (10)	0.0636 (11)	0.0412 (9)	−0.0057 (8)	0.0167 (8)	0.0005 (8)
N4	0.0426 (8)	0.0701 (10)	0.0471 (8)	0.0048 (7)	0.0173 (7)	0.0048 (7)

### Geometric parameters (Å, °)

**Table:**

N1—C7	1.3253 (16)	C8—C9	1.4823 (19)
N1—C15	1.3440 (18)	C9—C14	1.379 (2)
N2—C8	1.3356 (19)	C9—C10	1.381 (2)
N2—N3	1.3448 (17)	C10—C11	1.383 (2)
N3—C15	1.3322 (18)	C10—H10A	0.9300
C20—N4	1.3313 (18)	C11—C12	1.375 (2)
C20—C16	1.385 (2)	C11—H11A	0.9300
C20—H20A	0.9300	C12—C13	1.363 (2)
C1—C6	1.385 (2)	C12—H12A	0.9300
C1—C2	1.388 (2)	C13—C14	1.386 (2)
C1—H1B	0.9300	C13—H13A	0.9300
C2—C3	1.375 (2)	C14—H14A	0.9300
C2—H2B	0.9300	C15—C16	1.4788 (19)
C3—C4	1.370 (2)	C16—C17	1.386 (2)
C3—H3B	0.9300	C17—C18	1.377 (2)
C4—C5	1.381 (2)	C17—H17A	0.9300
C4—H4B	0.9300	C18—C19	1.371 (2)
C5—C6	1.386 (2)	C18—H18A	0.9300
C5—H5A	0.9300	C19—N4	1.335 (2)
C6—C7	1.4887 (19)	C19—H19A	0.9300
C7—C8	1.4126 (19)		
			
C7—N1—C15	116.31 (12)	C10—C9—C8	120.64 (13)
C8—N2—N3	119.33 (12)	C9—C10—C11	120.51 (15)
C15—N3—N2	118.20 (13)	C9—C10—H10A	119.7
N4—C20—C16	124.33 (15)	C11—C10—H10A	119.7
N4—C20—H20A	117.8	C12—C11—C10	120.12 (17)
C16—C20—H20A	117.8	C12—C11—H11A	119.9
C6—C1—C2	119.60 (16)	C10—C11—H11A	119.9
C6—C1—H1B	120.2	C13—C12—C11	119.82 (15)
C2—C1—H1B	120.2	C13—C12—H12A	120.1
C3—C2—C1	120.50 (16)	C11—C12—H12A	120.1
C3—C2—H2B	119.8	C12—C13—C14	120.27 (15)
C1—C2—H2B	119.8	C12—C13—H13A	119.9
C4—C3—C2	119.95 (16)	C14—C13—H13A	119.9
C4—C3—H3B	120.0	C9—C14—C13	120.54 (16)
C2—C3—H3B	120.0	C9—C14—H14A	119.7
C3—C4—C5	120.19 (16)	C13—C14—H14A	119.7
C3—C4—H4B	119.9	N3—C15—N1	125.41 (13)
C5—C4—H4B	119.9	N3—C15—C16	117.45 (13)
C4—C5—C6	120.33 (15)	N1—C15—C16	117.13 (12)
C4—C5—H5A	119.8	C20—C16—C17	117.00 (13)
C6—C5—H5A	119.8	C20—C16—C15	121.79 (13)
C1—C6—C5	119.42 (14)	C17—C16—C15	121.21 (13)
C1—C6—C7	120.15 (13)	C18—C17—C16	119.76 (15)
C5—C6—C7	120.41 (13)	C18—C17—H17A	120.1
N1—C7—C8	120.00 (13)	C16—C17—H17A	120.1
N1—C7—C6	117.05 (12)	C19—C18—C17	118.28 (15)
C8—C7—C6	122.95 (12)	C19—C18—H18A	120.9
N2—C8—C7	120.14 (13)	C17—C18—H18A	120.9
N2—C8—C9	116.00 (13)	N4—C19—C18	123.81 (15)
C7—C8—C9	123.86 (13)	N4—C19—H19A	118.1
C14—C9—C10	118.71 (14)	C18—C19—H19A	118.1
C14—C9—C8	120.64 (14)	C20—N4—C19	116.80 (14)

### Hydrogen-bond geometry (Å, °)

**Table:**

Cg1 and Cg2 are the centroids of the N4,C16–C20 and C1–C6 rings, respectively.

**Table:**

*D*—H···*A*	*D*—H	H···*A*	*D*···*A*	*D*—H···*A*
C20—H20A···N3	0.93	2.49	2.824 (4)	102
C13—H13A···Cg1^i^	0.93	3.49	3.345 (4)	91
C19—H19A···Cg2^ii^	0.93	3.67	3.109 (4)	121

Symmetry codes: (i) −*x*+1, *y*+1/2, −*z*+1/2; (ii) −*x*+1, −*y*+1, −*z*+1.
